# Capping the Cost of Compliance with the Kyoto Protocol and Recycling Revenues into Land-Use Projects

**DOI:** 10.1100/tsw.2001.70

**Published:** 2001-07-17

**Authors:** Bernhard Schlamadinger, Michael Obersteiner, Axel Michaelowa, Michael Grubb, Christian Azar, Yoshiki Yamagata, Donald Goldberg, Peter Read, Miko U. F. Kirschbaum, Philip M. Fearnside, Taishi Sugiyama, Ewald Rametsteiner, Klaus Boswald

**Affiliations:** Joanneum Research, A-8010 Graz, Austria

**Keywords:** climate change, greenhouse gases, CO_2_, offset certificates, price cap, price ceiling, carbon sequestration, deforestation, compliance, Kyoto Protocol

## Abstract

There is the concern among some countries that compliance costs with commitments under the Kyoto Protocol may be unacceptably high. There is also the concern that technical difficulties with the inclusion of land use, land-use change, and forestry activities in non-Annex I countries might lead to an effective exclusion of such activities from consideration under the Protocol. This paper is proposing a mechanism that addresses both these concerns. In essence, it is suggested that parties should be able to purchase fixed-price offset certificates if they feel they cannot achieve compliance through other means alone, such as by improved energy efficiency, increased use of renewable energy, or use of the flexible mechanisms in the Kyoto Protocol. These offset certificates would act as a price cap for the cost of compliance for any party to the Protocol. Revenues from purchase of the offset certificates would be directed to forest-based activities in non-Annex I countries such as forest protection that may carry multiple benefits including enhancing net carbon sequestration.

## INTRODUCTION

This paper addresses two concerns about the Kyoto Protocol: first, that its commitments might result in costs that are, at least for some parties, unacceptably high; and second, that technical difficulties with the inclusion of land use, land-use change, and forestry[a] activities in non-Annex I countries may lead to such activities being excluded altogether from consideration under the Protocol. The paper suggests a mechanism to place an upper bound on the cost of compliance, and thus to reduce uncertainty about the cost of ratification, and to direct revenues from the mechanism to forest-based activities that may carry multiple benefits including enhancing net $CO_2$ absorption. The paper shows that the proposed mechanism can simultaneously address a number of other issues that have contributed to the breakdown of negotiations in November 2000 in The Hague and subsequently, such as:

- how to deal with emissions that are in excess of the allocated quota under the Protocol (the "non-compliance" issue);
- concerns that the volume of credits from carbon sequestration would undermine the incentive for action on fossil-fuel emissions (the "scale" issue);
- the uncertainty and possible reversion of sink projects in the Clean Development Mechanism (CDM) (the "permanence" issue);
- the involvement of developing countries; and
- the environmental and social integrity of sequestration-related measures and their compatibility with other UN Conventions.

The proposal made here has two main objectives: to help limit the costs to Annex I countries of meeting their commitments under the Kyoto Protocol, and to help generate revenues for land-use projects that combine the aim of carbon sequestration with other locally important criteria for sustainable development in non-Annex I countries.

Correspondingly, we are suggesting here that parties should be able to purchase fixed-price *offset certificates* if they feel they cannot achieve compliance through other means. Revenues from these offset certificates would be directed to forest-based activities in non-Annex I countries. The paper confines itself to land-use projects that, for a variety of reasons, cannot or may not be included in the CDM. By improving prospects for compliance, providing revenues for forest enhancement/protection, and promoting sustainable development, the proposal is expected to have a positive effect on the global environment.

## CAPPING THE COST OF COMPLIANCE

The proposal is stimulated by the concept of capping the cost of achieving emissions reductions and thus compliance, an idea previously put forward, for example, by Resources For the Future[1] and by Victor[2]. It is implemented here by allowing governments to purchase "offset certificates". Governments that find that they are at risk of not meeting their commitments could make up the short-fall by purchasing offset certificates, which would be counted as equivalent to genuine emission reductions, and would be issued at a fixed, predetermined price.

This would mean that governments could calculate, before ratifying the Kyoto Protocol, the maximum cost of compliance. The availability of offset certificates at a fixed price would function as a "safety valve" to avoid the risk of very high costs to individual economies. The

[a] The term land use, land-use change, and forestry is frequently abbreviated as LULUCF. In this paper we take the term "sequestration" to mean any activities that reduce greenhouse-gas emissions from, or enhance removals of greenhouse gases by, land-use systems.

price would have to be uniform across countries, i.e., negotiated internationally to prevent countries from setting a too low domestic price, thus unreasonably reducing its compliance costs and possibly encouraging other countries to meet Kyoto targets through extensive emissions trading with countries that issued offset certificates. With unlimited emissions trading, the lowest national certificate price would constitute the effective international price.

Fixed-price offset certificates could be issued at any time.[b] If the market price of greenhouse-gas emission allowances derived from the Kyoto system is low, there would be few buyers for the offset certificates. If, however, the traded $CO_2$ price reaches the certificate price, then there could be significant demand.

The price of offset certificates is thus a crucial variable. If it is too low, then there will be little investment in energy systems and technological innovation, and extensive purchase of offset certificates. If it is too high, then there will be no revenue and no cost limitation. An optimum certificate price would protect against market prices rising to a level at which countries would find compliance to be too onerous, while still providing sufficient incentives for projects, policies and measures, and technological development to reduce or limit emissions.

## BUYERS OF OFFSET CERTIFICATES

There could be two types of buyers of offset certificates:

- Private-sector companies that want to achieve compliance with whatever domestic implementation of the Kyoto Protocol they face. Any offset certificates obtained by them would count toward international compliance by parties to the Protocol, as explained above. This requires that the offset certificates be accepted by governments, i.e., the parties to the Kyoto Protocol, as equivalent to emission allowances created by the Protocol ("assigned amounts", in Kyoto parlance). For example, if company X in country A obtains offset certificates amounting to 10,000 tons of $CO_{2equ}$, then the same quantity would be added to the assigned amount of the country in which company X operates.
- Governments that are still heading toward non-compliance after having 1) implemented domestic policies and measures, 2) used the flexible mechanisms (Joint Implementation, CDM, emissions trading) to acquire emission permits from abroad, and 3) accounted for certificate-purchasing by the private sector. In this case, in essence governments would sell these new permits to themselves. They would take the extent of their likely non-compliance, convert this into monetary units according to the established certificate price, and commit this amount of money to projects as outlined below.

## RECYCLING OF THE REVENUES INTO SEQUESTRATION PROJECTS IN NON-ANNEX I COUNTRIES

The proceeds from the sale of these offset certificates would be recycled into land-use projects that increase net $CO_2$ sequestration[c] but may not otherwise be eligible under the Kyoto rules. The countries that issued the offset certificates would get assigned amount units (AAUs) for each offset certificate and have to prove that revenues are recycled into sequestration projects

[b] Offset certificates could be issued by the government of the country where the purchaser is located. They could also be issued by an international agency. The latter would limit the influence of individual countries regarding where, and in what types of projects, the investments occur but it could offer additional assurance that the carbon benefits claimed are real.

[c] The use of sequestration projects as a "safety valve" has been proposed, independently of this study, by Taishi Sugiyama[4]. His design was an elaboration of the "Climate Change Fund" in relation to non-compliance with the Kyoto Protocol. The Climate Change Fund appeared in negotiating text (FCCC/CP/2000/5/add.3).

respecting internationally agreed criteria. Hence, it would remove the temptation of the issuing country to maximize carbon benefits without regard for other environmental and socio-economic considerations.

There is currently debate about whether any land-use projects should be allowed to qualify for credits under the Protocol's Clean Development Mechanism. Deforestation in non-Annex I countries is the single largest emissions source that is not addressed by the Kyoto Protocol (average emissions of 1600 MtC (5900 $MtCO_2$)/yr in the 1990s with an uncertainty range of +/- 800 MtC (2900 $MtCO_2$)/yr[13]). Including 'avoided deforestation' for such crediting has been controversial. It is perceived that it would create a 'moral hazard': countries may have an incentive to threaten to cut down forests in order to maximize $CO_2$ credits for not doing so.[d] There is also concern about the uncertainties inherent in establishing "without project" baselines for deforestation. In addition, there is the more general issue of possible non-permanence of the net carbon stored in any type of sequestration project[3].

If, due to such difficulties, these activities are not included for crediting under the CDM, then incentives for improved land management in developing countries would risk being excluded altogether from the Kyoto system. We propose instead that revenues from the offset certificates should be channeled into such activities.[e] Because the incentive would not be purely to maximize carbon sequestration, many of the problems associated with crediting land-use activities would be reduced or removed. It would then be far easier to strike a balance between carbon uptake and the many other objectives of land-use management, such as protecting and enhancing biodiversity, protecting watersheds, reducing soil erosion, or improving local livelihoods.

Nevertheless, many such projects would likely yield net carbon sequestration at per-ton costs that are below the price of offset certificates,[f] so that for each additional ton of emissions in Annex I countries, made possible through issuance of an offset certificate, more than one ton of emission reduction or sink enhancement would occur in non-Annex I countries. Insofar as this is the case, the net $CO_2$ reduction could even exceed the Kyoto target. The exact amount of emission reductions could not be determined beforehand, but would depend upon the overall quality, characteristic, and cost of the projects funded.

For illustration, it is assumed that the CDM includes sequestration activities in a very limited way if at all (for example, restricted to afforestation and reforestation activities). The revenues from sale of offset certificates would be invested in a portfolio of sequestration projects that are not part of the CDM, including possibly afforestation and reforestation projects to reestablish natural forests, forest protection, and forest restoration.[g]

The allocation of funds to the projects would be carried out by each certificate-issuing entity. A mechanism of competitive bidding or auctioning[1,7] is recommended. For the bidding a number of eligibility and allocation criteria could be used in addition to the price per ton of $CO_2$. Such criteria are deemed to be necessary to ensure environmental and social integrity of projects and compatibility with other UN Conventions such as the Convention on Biological Diversity.

[d] The adoption of historical deforestation baselines has been proposed to avert this perceived moral hazard. See, e.g., Goldberg[5].

[e] Note that the latest proposed framework[6] includes a provision that part of the so called "adaptation fund" be used to "[implement] measures regarding forest conservation, rehabilitation of degraded land, and [combat] desertification, particularly in Africa". The present proposal explores and elaborates upon this suggestion.

[f] The first few projects may be available at a low cost. As demand increases, the cost will also increase. The volume of $CO_2$ mitigation in non-Annex I sequestration projects at any given cost is not well known. Once the cost approaches the certificate price and the volume of reasonable sequestration projects is used up, other re-investment channels could be used such as a geographical distribution fund (CDM projects in locations where they are otherwise less likely; Kevin Baumert, pers. communication) or an energy technology fund. Alternatively, it would be possible to allow energy-type CDM projects to compete for the revenues available from offset certificates.

[g] Some revenues could be focused to protect "hot spots", i.e., areas which are very rich in biodiversity[8]. The selection of the project locations would be up to the individual governments that issue offset-certificates. For example, Brazil could offer various deforestation avoidance projects; India might offer reforestation. The German government could decide whether it wanted to buy high-biodiversity Brazilian forest protection projects, or Indian land improvement, or some combination.

Criteria and indicators for the sustainable management of forest resources have been elaborated in recent years by a number of international fora and initiatives. Further, the projects would also need to comply with the criteria used in the CDM, such as additionality.

This mechanism is similar to the Emission Reduction Unit Procurement Tender (ERUPT) conducted by the Dutch government for Joint Implementation and planned for later in 2001 also for CDM projects (www.senter.nl/erupt). The ERUPT program guarantees the most economic outcome for the investor, while taking into account other important criteria. Any excess of the certificate price over the strike price in the auction would constitute a gain that can be invested into additional projects, thus ensuring that more $CO_2$ is sequestered than the additional emissions permitted by the issued offset certificates. In this sense, it would lead to over-compliance if sequestration projects in non-Annex I countries yield reductions at a cost below that of “Kyoto-eligible” projects that would have been implemented in absence of the fixed certificate price.[h]

**Example 1: $CO_2$ price cap with revenue recycling into sequestration projects – how would it work?**

| | |
|---|---|
| 1990 | Country X reports emissions of 100 $MtCO_2$/yr |
| 1997 | The Kyoto Protocol requires Country X to reduce emissions to 90 $MtCO_2$/yr during the first commitment period. The estimated “business as usual” emissions are 120 $MtCO_2$ / yr. |
| 2002 | Country X starts to reduce emissions through domestic policies and measures (for example, greater use of renewable energy and improved energy efficiency), and investment in Joint Implementation and CDM projects. The effort results in an emission reduction of 10 $MtCO_2$/yr for each of the options (total of 20 $MtCO_2$). Another 5 $MtCO_2$ can be claimed through carbon sequestration. Detailed studies show that further emission reductions come at costs between 30 and 50 US$/$tCO_2$. |
| 2010 | Country X issues 5 million offset certificates (one per $tCO_2$) at a certificate price of 20 US$ per ton of $CO_2$. The country uses the revenues, which add up to 100 million US$, to issue an international tender for forest protection projects in non-Annex I countries. These projects have to fulfill additional qualifier criteria, such as biodiversity protection or enhancement, socio-economic considerations, etc. Therefore, the emission reduction is lower than in projects that do not have to comply with such standards. Carbon emissions avoided: 20 $MtCO_2$ (average cost at 5$ / $tCO_2$). Without the very stringent “non-carbon” criteria, the projects may only have cost 3$ / $tCO_2$. |
| 2013 | While country X has exceeded its original emissions limit by 5 $MtCO_2$, an additional amount of 20 $MtCO_2$ has been saved in forest protection. Third-party verification shows that the uncertainties surrounding this estimate are large, and only 10 $MtCO_2$ can be considered a reduction at 90% confidence. Furthermore 3 $MtCO_2$ are at risk of being lost to the atmosphere at a later stage (non-permanence). However, the remaining 7 $MtCO_2$ are still sufficient to offset the additional emissions of 5 $MtCO_2$ from country X. The host country would guarantee the maintenance of the “protected” carbon stocks for a predetermined time period. |

[h] This is true to the extent that the sequestration projects are excluded from crediting under the Kyoto Protocol, because projects already eligible would have been taken up by the market before the cap price is reached. Forest projects in the CDM are, at the time of writing, limited to afforestation and reforestation (subject to addressing issues of non-permanence, additionality, leakage, scale, uncertain-ties, socio-economic, and environmental impacts at COP8[6]).

## CERTIFICATE PRICE SUBJECT TO A DISCOUNT RATE

With a certificate price that remains constant, market participants are likely to wait until the last moment (i.e., the end of the first commitment period) to buy offset certificates. This is to be expected because 1) there is a chance that credits can still be obtained at a price lower than the certificate price, and 2) in terms of net present value it is economical to purchase the offset certificates as late as possible. If offset certificates are purchased only at the end of the first commitment period, then all investment in sequestration projects in developing countries would be delayed and occur within a very short time beginning in 2013, resulting in large price uncertainties. Also, it may be difficult to initiate a large number of projects in a short time unless governments issuing the offset certificates anticipate eventual revenues and smooth the flow of projects with funds from their general revenues. Without such smoothing, speculation about the large funds that would become available in the end could lead to even lower levels of early abatement in this sector than would have been the case without this mechanism. This could also have negative effects on the “business as usual” baseline development which would eventually be used as a benchmark for determining additionality of the sequestration projects initiated after the first commitment period.

Apart from these difficulties, there are other benefits for the atmosphere and for sustainable development objectives in undertaking mitigation activities, for example forest protection, earlier rather than later. The original proposal for a “Clean Development Fund”, made by Brazil during the negotiations in Kyoto, would have allowed investment to occur only after the first commitment period. In response to this proposal it was argued that it would be preferable to allow investment already before the end of the first commitment period, and this resulted in the Clean Development Mechanism, as elaborated in Article 12 of the Kyoto Protocol.

These arguments speak in favor of issuing offset certificates before the end of the first commitment period and making the purchase of such offset certificates attractive through discounting of the certificate price with time. One argument *against* early issuance of offset certificates, and a discounting of the certificate price, is that the mechanism should be used as a safety valve, not as a source of cheap permits. Assuming that the market price of emission allowances may fluctuate during the first commitment period, one may not want countries to rush to the mechanism at the first sign of high market prices. Also, it has been argued that perhaps a time-discount for the certificate price may not be warranted because of price levels that would be too low in the early years. However, even without a discounting applied to the certificate price, the maximum price of AAUs and other traded emissions permits would follow an implicit discounted curve due to their fungibility with the offset certificates and due to the availability of bank finance.

Figure 1 shows the discounted certificate price with the assumption of an average price, during the first commitment period, of US\$20/t$CO_2$ (about \$75/tC). The discount rate would have to be high enough to encourage market participants to buy offset certificates before the end of the first commitment period, especially if they are likely not to be in compliance. The discount rate should therefore not be much lower than the market interest rate, because otherwise early investment would not occur. However, the discount rate should not be higher than the market interest rate to avoid making it profitable to borrow money for buying offset certificates. In Fig. 1 the discount rate was assumed to be 6%.

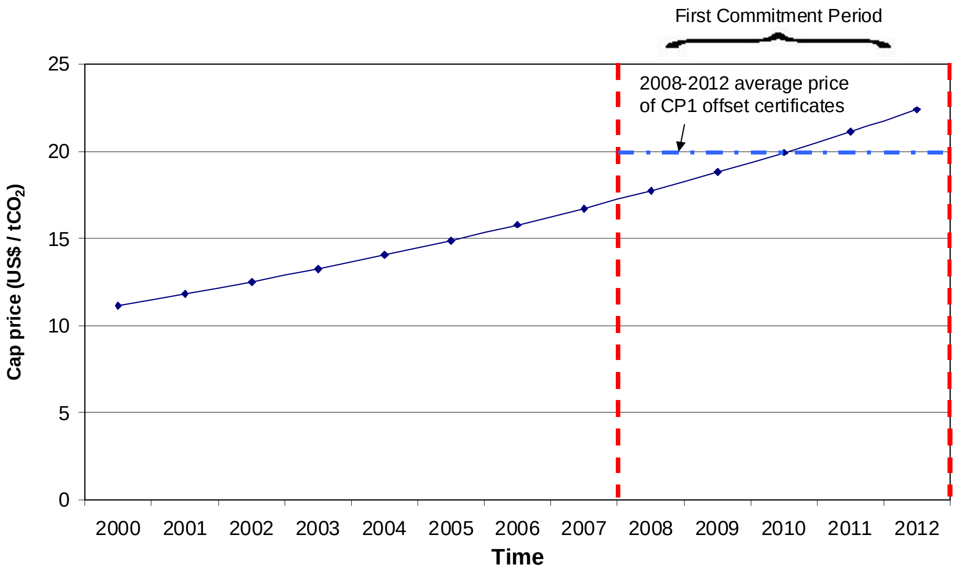

FIGURE 1. Increasing certificate price (CP1 = first commitment period).

## HOW DOES THIS PROPOSAL ADDRESS THE VARIOUS CONCERNS LISTED AT THE OUTSET OF THIS PAPER?

### Uncertainties about Costs of Implementing Kyoto

The total cost of Kyoto implementation and the economic risk of ratifying the Protocol would be limited. This, however, would necessitate that the certificate price be negotiated before ratification by those parties that contemplate using offset certificates.

### The Non-Compliance Issue

Because purchase of fixed-priced offset certificates would be available to any Party that is otherwise failing to comply with its emission limitation commitment, the certificate price would constitute an analog to a "non-compliance fee", except that it would require the establishment of, and verification structure for, mitigation projects.

### Concern that the Volume of Credits from Carbon Sequestration would Undermine the Incentive for Action on Fossil-Fuel Emissions

The issues of scale for sequestration projects would be addressed because the offset certificates would only become available once the market price for $CO_2$ emission credits reached the certificate price. The level of that price would determine the quantity of offset certificates that would be purchased. Setting a relatively high certificate price would ensure that a reasonable amount of mitigation is already occurring in the energy sector before this mechanism is invoked. Due to the cost limitation brought about by the 'safety valve', reasonably restrictive regulations could be adopted for sink projects under the CDM.[i] The mechanism proposed here would also ensure against underuse of sequestration projects in developing countries.

### Uncertainty and Possible Non-permanence of Sequestration Projects

It is expected that the revenue from each offset certificate (sold to the equivalent of 1 $tCO_2$ at the certificate price) will yield several tons of actual net carbon sequestration in sink projects. Therefore, considerations of uncertainty, permanence and leakage are less critical. For example, if each offset certificate yields two tons of $CO_2$ emission reductions, then the failure of half of these projects due to uncertainty, leakage, or non-permanence, would still not increase Annex I emissions above the overall target of the Kyoto Protocol. Because the actual emission reductions resulting from projects would not need to be as tightly measured and monitored as they would in the CDM, the proposed mechanism also avoids vested interests — especially by the purchasing party — to inflate expected emissions reductions.

### Involvement of Developing Countries

Developing countries are contributing toward reaching the Kyoto targets by providing mitigation opportunities through the CDM. The use of revenues from the issuance of the offset certificates could further enhance the role of these countries in the area of 'land use'. In this area they could contribute comparatively more if additional mitigation activities could be encouraged (particularly addressing emissions from deforestation), and if there were a commitment for preserving the carbon benefits for a defined time period[9].

## OUTLOOK TO THE FIRST COMMITMENT PERIOD

Whether, and how many, offset certificates would be issued, depends on the demand for emission allowances, and thus importantly on U.S. participation. Some scenarios would be as follows:

1. Participation by the USA from the beginning, leading to high demand for credits. The global demand for emission allowances is estimated to be on the order of 3000 to 4000 $MtCO_2$/year, about 1000 of which may be attained by trading of surplus allowances from Economies in Transition ("hot air", in Kyoto parlance)[10,11].
2. Implementation of the Kyoto Protocol without the U.S., leading to lower demand and a lower market price of $CO_2$ emission credits – probably far below any sensible price cap unless surplus trading is avoided.[j] The demand for emission allowances is estimated to be on the order of 1000 $MtCO_2$/yr which could be fully covered by surplus allowances from Economies in Transition[10,11].
3. Same as scenario 2, but the U.S. joins the system later. If there is an expectation that this will happen, the $CO_2$ price could rise to the level of the certificate price.

At the time of this writing (early July 2001) indications are that the European Union (together with the "Economies in Transition" in Eastern Europe), Russia, and Japan might ratify the Kyoto

[i] Negotiating text at the time of writing proposes to include in the CDM afforestation and reforestation, subject to addressing modalities for dealing with non-permanence, additionality, leakage, scale, uncertainties, and socio-economic and environmental impacts (including impacts on bio-diversity and natural ecosystems)[6].

[j] Recognizing that unlimited sales of its surplus allowance would lead to extremely low prices, Russia is discussing with other parties approaches that could sustain appropriate prices through mechanisms that would guarantee environmentally productive use of the revenues[12].

Protocol thus bringing it into force, but the U.S. might not ratify it at this time. This would correspond to scenarios 2 or 3, depending on further action by the U.S.

The elements of the proposal made here could be added to the current framework[6] at a later time. That is, agreement on a $CO_2$ price cap, and on recycling of the revenues into currently not-eligible sequestration projects could be reached later, if and when the demand for emission allowances is sufficiently large — for example, due to ratification by additional countries.[k] However, as already mentioned, this addition and the certificate price would have to be negotiated before countries ratify the Protocol if this measure is to have its full benefit in allaying fears of prohibitive expense.

## CONCLUSIONS

We propose that a price cap be employed to limit the costs of Kyoto compliance. Issuing offset-certificates at the cap price, with revenues recycled into land-use activities in non-Annex I countries that otherwise would not be counted under the Kyoto Protocol could address a number of the difficulties that have contributed to the uncertain future of this treaty. By addressing these difficulties, and in particular by addressing stated concerns by the U.S. about cost and the likely exclusion of certain carbon-sink categories, the approach could both lead to a re-evaluation of the merits of the Kyoto Protocol on the part of nonparticipating countries as well as providing funds for multi-purpose land-use projects in non-Annex I countries.

## ACKNOWLEDGMENT

We are grateful for invaluable comments received from Gregg Marland (Oak Ridge National Laboratory, United States), Kevin Baumert (World Resources Institute, United States), Roger Sedjo (Resources For the Future, United States), David Victor (Council on Foreign Relations, United States), Hans Nieuwenhuis (Ministry of the Environment, The Netherlands), Gerald Kapp (GFA Terra Systems GmbH, Germany), Naomi Pena (Pew Center on Global Climate Change, United States), and from two anonymous reviewers. This paper reflects the views of the authors and not necessarily the views of the institutions with which they are affiliated.
